# “Catch, Flip, and Remove” Technique for Retrieval of a Hypermobile Detached Leadless Pacemaker: A Case Report and Literature Review

**DOI:** 10.19102/icrm.2022.130404

**Published:** 2022-04-15

**Authors:** Michael Co, Ravi Mandapati, Jason Smith, Kyle Cooper, Jalaj Garg, Tahmeed Contractor

**Affiliations:** ^1^Department of Cardiology, Cardiac Arrhythmia Service, Loma Linda University Medical Center, Loma Linda, CA, USA; ^2^Department of Interventional Radiology, Loma Linda University Medical Center, Loma Linda, CA, USA

**Keywords:** Complication, dislodgement, extraction, leadless pacemaker, snaring

## Abstract

Leadless pacemaker (LP) detachment is a rare but life-threatening complication that may occur during implantation. While different snaring techniques have been described to remove partially or completely detached LPs, there are currently no reports of snaring a hypermobile LP that travels between different cardiac chambers. This report describes a technique to successfully snare a hypermobile detached LP by first “catching” onto the tines for stabilization with the help of a multi-loop snare, followed by using a second snare for the proximal retrieval feature.

## Introduction

Leadless pacemaker (LP) detachment is a rare, potentially life-threatening complication. While different snaring techniques have been described to remove partially or completely detached LPs,^1–3^ there are currently no reports of snaring a hypermobile LP that travels between different cardiac chambers.

This report describes a technique to successfully snare a hypermobile detached LP by first “catching” onto the tines for stabilization with the help of a multi-loop snare, then using a second snare for the proximal retrieval feature (PRF). We also discuss in detail and summarize various techniques for the removal of partially or completely detached LPs.

## Case report

A 64-year-old man with a past medical history of end-stage renal disease (hemodialysis via left arteriovenous fistula), permanent atrial fibrillation (AF), and ischemic cardiomyopathy (left ventricular ejection fraction of 25%) presented to our hospital with methicillin-resistant *Staphylococcus aureus* bacteremia. He had a right-sided biventricular (BiV) implantable cardioverter-defibrillator (ICD) that was implanted 5 years ago. In view of his persistent bacteremia (and no other identified source), complete system removal was deemed appropriate. Due to his underlying comorbid conditions as well as issues with access (right-sided infection, left-sided fistula), re-implantation of a transvenous device with leads (such as a biventricular pacemaker or ICD) was deferred after a detailed discussion. To minimize intravascular hardware, after using a shared process of decision-making, it was decided an LP would be placed for pacing support and a future subcutaneous ICD would be inserted to reduce the risk of death from ventricular arrhythmias after extraction. LP implantation had been safely performed at the time of lead extraction in a reported series with a low risk of recurrent infections or LP dislodgement.^4^ Given that our patient had complete heart block with no underlying ventricular escape rhythm (even noted on prior outpatient device interrogation) and to potentially avoid an additional procedure, an LP (Micra™ Transcatheter Pacing System; Medtronic, Minneapolis, MN, USA) was implanted at the time of extraction. The final threshold was 1.7–2.0 V at a pulse width of 0.24 ms, which was felt to be acceptable given that several sites were attempted prior to the final position. The right ventricular aspect of the lead and the LP were distant from each other. The BiV ICD system was then successfully extracted, and no interaction was noted between the LP and right ventricular lead or extraction sheath. The threshold was unchanged immediately after the procedure. However, over the course of his hospitalization, the patient showed rising thresholds. Even after programming the highest output, intermittent loss of capture was seen, necessitating placement of a temporary pacemaker. As there was no plan to re-implant another transvenous device requiring leads in the future, he was brought back for implantation of a new LP 8 days later.

**Figure 1:**
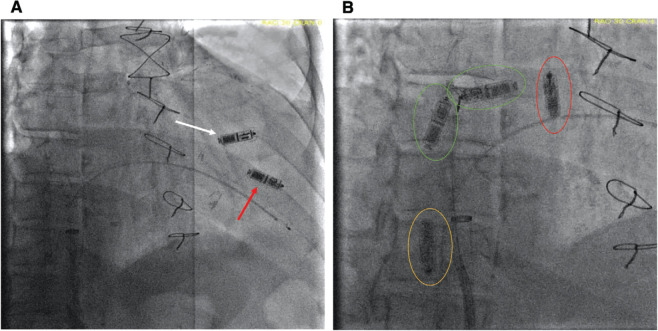
**A**: Fluoroscopic image illustrating a new leadless pacemaker (white arrow) attached superiorly to the prior one (red arrow); **B**: Overlapped fluoroscopic images from the same series illustrating the device floating between the inferior vena cava (yellow circle), right atrium (green circle), and tricuspid valve annulus (red circle).

**Figure 2:**
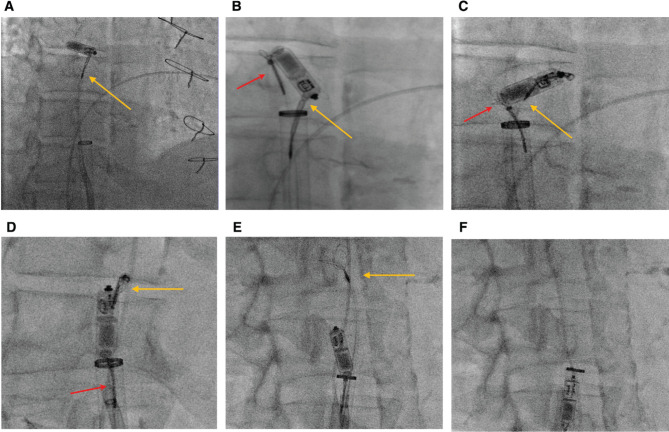
Two-snare technique for leadless pacemaker removal. **A**: Multi-loop Merit EN Snare^®^ snare (yellow arrow) was used to capture the tines. **B**: Multi-loop Argon Atrieve™ snare (red arrow) was used to snare the proximal retrieval feature. **C and D**: Using a push (yellow arrow)–pull (red arrow) method between the 2 snares, the leadless pacemaker was ultimately positioned such that it was coaxial with the sheath. **E**: The EN Snare^®^ (yellow arrow) snare was released. **F**: The Atrieve™ snare was used to pull the pacemaker into the delivery sheath.

Given the possibility of dislodgement of the temporary pacemaker during attempted removal of the old LP and the fact that it is currently not feasible to reuse a dislodged LP, we decided that the benefit of placing a new LP first for stable pacing support outweighed the risk of dislodging it while retrieving the old LP. Following implantation of a new LP in a higher position, an attempt was made to snare the previous LP **(Figure 1A)**. Using the 27-French (Fr) Micra™ delivery sheath with an 8.5-Fr Agilis sheath (Abbott, Chicago, IL, USA) inside it, a 12–20-mm multi-loop snare (EN Snare^®^; Merit Medical, South Jordan, UT, USA) was used to snare the LP. However, after snaring the body of the LP and while trying to move the snare back to the PRF, the LP broke loose and began moving freely between the inferior vena cava (IVC), right atrium (RA), and tricuspid valve annular areas **(Figure 1B)**. Given the free-floating nature of the device, we decided to use a 2-snare technique that we implemented as follows:

1. **“Catching” the tines.** Using the existent system (27-Fr delivery sheath with an 8.5-Fr Agilis sheath inside it), the same 12–20-mm multi-loop snare was placed in the RA with the loops open and “fishing” for the freely moving LP. Once a loop caught one of the tines, the snare was tightened to stabilize the LP pacemaker **(Figure 2A)**. The LP was then brought closer to the open edge of the delivery sheath.
2. **Snaring the PRF.** As the long axis of the LP was perpendicular to the long axis of the delivery sheath, as well as with tenuous control of the device snared by a single thin tine, we decided to place a second multi-loop snare (Atrieve™ multi-loop snare; Argon Medical Devices, Frisco, TX, USA) through the same delivery sheath (adjacent to the Agilis sheath) to snare the PRF of the LP. This snare was advanced in the open position beyond the LP and then pulled back to snare the PRF **(Figure 2B)**.
3. **Re-orientation of the LP.** Once the PRF was secured using the second snare, a “push–pull” method was employed, during which the first snare was pushed forward while pulling back the second snare to flip the orientation of the LP and point the PRF toward the opening of the delivery sheath **(Figure 2C and D)**. During this maneuver, the entire system was also gradually brought down into the IVC to facilitate easier re-orientation in the tubular IVC compared to the RA.
4. **Release of tine snare/LP retrieval.** Once the PRF was pointed toward the opening of the delivery sheath, the first snare was loosened and carefully advanced to disengage it from the tine and then pulled back into the delivery sheath along with the Agilis sheath **(Figure 2E)**. The second snare was then used to pull the LP into the delivery sheath **(Figure 2F)**. The sheath and device were then removed, and hemostasis at the sheath entry site was obtained by a figure-of-8 suture.

The patient tolerated this procedure without any complications, with stable thresholds on the new device check. He was discharged to a nursing facility on a 6-week course of antibiotics on postoperative day 3.

## Discussion

We report a 2-snare technique used to retrieve a hypermobile free-floating LP using multi-loop snares. An LP that is free-floating between the right-sided cardiac chambers and the IVC has hitherto not been reported, and there is limited available literature to guide the management of detached LPs.

LPs have evolved as an important tool in the armamentarium for the management of brady-arrhythmias. It is especially useful in patients with permanent AF who require ventricular pacing and are also at high risk of infection, such as the patient reported here. However, a potential complication is LP detachment, with an incidence of 0.06% in the Micra™ Post-approval Registry.^5^ Detachments can either be partial due to attachment of a single tine (“cliffhanger LP”) or complete. Partial detachment can be life-threatening in a pacemaker-dependent patient if capture is lost and can progress to complete detachment that can either result in a free-floating and hypermobile device or embolization of the LP to a remote location, such as the pulmonary artery. When hypermobile, the detachment can theoretically lead to ventricular arrhythmias or damage to intracardiac structures such as the tricuspid valve, papillary muscles, and chordae tendinae. Immediate management of this complication is therefore crucial.

The 2-snare technique for LP retrieval has been described in the literature and is summarized in **Table 1**.^1–3^ The first case, reported by Goyal et al.,^1^ involved a patient with an initial partially detached LP that became completely dislodged prior to a capture. A single-loop snare was used for the body, but the perpendicular orientation of the LP to the delivery sheath precluded removal. A second single-loop snare was used to capture the PRF, followed by removal of the first snare and retrieval of the LP. The second case, reported by Hasegawa-Tamba et al.,^2^ also involved a partially detached rather than free-floating device. After failing to catch the swaying device with a single-snare technique, a 2-directional snare technique via the IVC (using a 23-Fr Micra introducer sheath) and superior vena cava (using a 9-Fr sheath) was used to retrieve the wobbling device successfully. The superior multi-loop snare stabilized the tines, while the inferior single-loop snare was used for the PRF and device removal. A third case reported by Kawasaki et al.^3^ describes the removal of the free-floating device in the RA using 2 single-loop snares through 2 different access sites. After stabilization of the tines with the first snare via the delivery sheath, a second snare from a separate access site was used for the PRF. The first snare was then released and used to grasp the neck of the second snare, followed by removal.

Our technique introduces new improvements to the techniques used in the prior cases. In contrast to the case by Goyal et al.^1^ (where it is unclear if the LP was traveling between cardiac chambers), we used multi-loop snares to increase the likelihood of securing the free-floating LP in the shortest amount of time. This is particularly important to reduce the risk of embolism and damage to surrounding structures, which can happen relatively quickly. Compared to the procedures of Hasegawa-Tamba et al.^2^ and Kawasaki et al.,^3^ our second snare was introduced through the same sheath, saving time and reducing the risks associated with 2 access sites. Our technique did not involve releasing the first snare that captured the body of the LP to make the LP coaxial to the introducer sheath (as described by Goyal et al.^1^) or snaring the neck of the second snare to avoid unintentional release of the second snare prior to retrieval (as described by Kawasaki et al.^3^). Instead, we used a simpler, push–pull technique (described previously) to achieve the same result. The benefit of the push–pull technique is the avoidance of the chance that the device becomes dislodged again while trying to reorient the device. This may not be possible in all patients, especially in those with smaller hearts, and larger studies in different patient populations are required to confirm the universal feasibility of this technique.

In conclusion, a hypermobile, detached LP was percutaneously removed using a 2-snare technique to first “catch” the tines and stabilize the device, followed by alignment (“flip”) and retraction into the 27-Fr delivery sheath.

## Figures and Tables

**Table 1:** Available Reports on the Removal of Detached Leadless Cardiac Pacemakers

Author/Year	Partial or Complete Detachment	Free-floating?	Approach Type(s) and Number of Access Sites	Types of Snares	Technique
Goyal et al.^1^	Partial^a^	No	Inferior approach with single access site	Single-loop snares	First snare for body and second snare for PRF; first snare released prior to alignment/removal
Hasegawa-Tamba et al.^2^	Partial	No	Inferior and superior approaches with 2 access sites (1 superior and 1 inferior)	Single- (inferior) and multi-loop (superior) snare	Superior snare to hold the tines; inferior snare for PRF and removal
Kawasaki et al.^3^	Complete	Yes^b^	Inferior approach with 2 access sites	Single-loop snares	First snare used for tines and second snare used for PRF; first snare was then used to catch the “neck” of the second snare

*Abbreviation:* PRF, proximal retrieval feature.

^a^ Completely detached after attempted snaring.

^b^ Free-floating in the RA only.
